# Changes in clinical disease activity are weakly linked to changes in MRI inflammation on treat-to-target escalation of therapy in rheumatoid arthritis

**DOI:** 10.1186/s13075-017-1433-7

**Published:** 2017-10-24

**Authors:** Fiona M. McQueen, Peter Chapman, Terina Pollock, Dena D’Souza, Arier C. Lee, Nicola Dalbeth, Lisa Stamp, Karen Lindsay, Anthony Doyle

**Affiliations:** 1 Department of Molecular Medicine and Pathology, Faculty of Medical and Health Sciences, The University of Auckland, Private Bag 92019, Auckland, New Zealand; 2 Department of Radiology, Auckland City Hospital, 2 Park Rd, Grafton, Auckland, 1023 New Zealand; 3 Section of Epidemiology & Biostatistics, School of Population Health (Tamaki Campus), University of Auckland, Auckland, 1142 New Zealand; 4 Bone & Joint Research Group, Department of Medicine, The University of Auckland, Private Bag 92019, Auckland, New Zealand; 5 Department of Rheumatology, Greenlane Clinical Centre, Auckland District Health Board, 214 Green Lane West, Epsom, Auckland, 1051 New Zealand; 6 Department of Rheumatology, Immunology & Allergy, Christchurch Hospital, PO Box 4710, Christchurch, 8140 New Zealand; 7 Department of Medicine, University of Otago, PO Box 4345, Christchurch, 8140 New Zealand

## Abstract

**Background:**

Rheumatoid arthritis (RA) treat-to-target (T2T) regimens often use the disease activity score (28 joints) incorporating C-reactive protein (DAS28_CRP_) as an outcome measure. We compared changes in the DAS28_CRP_ with changes in magnetic resonance imaging (MRI) inflammation on treatment escalation.

**Methods:**

Eighty seropositive RA patients with active disease were enrolled. Group A (*N* = 57) escalated to another conventional disease-modifying therapy (cDMARD) combination, and Group B (*N* = 23) to anti-TNF therapy/cDMARDs. Contrast-enhanced 3T-MRI wrist scans were obtained before and 4 months after regimen change. Scan pairs were scored for inflammation (MRI(i)) and damage. Disease activity was assessed using the DAS28_CRP_.

**Results:**

Eighty patients were enrolled and 66 MRI scan pairs were available for analysis. Intra-reader reliability was high: intraclass correlation coefficient (average) 0.89 (0.56–0.97). ΔDAS28_CRP_ did not differ between groups: Group A, −0.94 (−3.30, 1.61); Group B, −1.53 (−3.59, 0.56) (*p* = 0.45). ΔMRI(i) also did not differ: Group A, 0 (−25, 10); Group B, −1 (−15, 28) (*p* = 0.12). Combining groups, ΔMRI(i) correlated weakly with ΔDAS28_CRP_ (Spearman’s 0.36, *p* = 0.003). Using multiple linear regression analysis adjusting for confounders, ΔDAS28_CRP_ was associated with ΔMRI(i) (*p* = 0.056). Of the individual MRI measures, only Δtenosynovitis correlated with ΔDAS28_CRP_ (Spearman’s 0.33, *p* = 0.007). ΔMRI(i) was negatively associated with the MRI erosion score at entry (*p* = 0.0052).

**Conclusions:**

We report the first study investigating the link between changes in clinical and imaging inflammation in a real-world RA cohort escalating to conventional and biologic DMARDs. The association was significant but relatively weak, suggesting that MRI targets cannot yet be advocated as outcomes for T2T escalation.

**Trial registration:**

ANZCTR 12614000895684. Registered 22 August 2014.

## Background

Rheumatologists routinely manage rheumatoid arthritis (RA) using a treat-to-target (T2T) regimens [1], escalating therapy in patients who fail to reach remission or a low disease activity state [2]. Management is often initiated with methotrexate alone and then escalation proceeds to combination therapy with conventional disease-modifying anti-rheumatic agents (cDMARDs) and biologic DMARDs (bDMARDs). In New Zealand, cDMARD combinations include triple therapy (methotrexate, sulphasalazine, hydroxychloroquine) and methotrexate/leflunomide, although other options are available. The New Zealand Pharmaceutical Management Agency (PHARMAC), responsible for buying and funding pharmaceuticals, has defined criteria for funding bDMARDs including a trial of three different cDMARD combinations [3]. For those with an inadequate response to cDMARDs, escalation to methotrexate/anti-tumour necrosis factor (anti-TNF) agents follows and subsequently other bDMARDs. Adequacy of response is usually measured by the DAS28_CRP_ [4, 5].

MRI is a sensitive imaging modality that can reveal articular inflammation and joint damage [6, 7]. The OMERACT RA MRI score (RAMRIS) [8] and the Harvaardsholm tenosynovitis score [9] can quantify inflammation in a relatively reproducible manner. Studies show that MRI can detect more inflammation than physical examination. Up to 96% of asymptomatic RA patients with normal joints have MRI synovitis and 46% have MRI bone oedema/osteitis [10]. Krabben et al. [11] found MRI inflammation in 66% of non-swollen wrist joints in patients with early RA. Severe grades of bone oedema (osteitis) were common in non-swollen joints. We and others have found that osteitis is a predictor of joint erosion [12, 13]. Although MRI is more sensitive than clinical assessment for detection of joint inflammation and damage, MRI outcomes are not yet part of accepted clinical practice because of feasibility issues including cost and accessibility. It is also not clear whether using the change in MRI inflammation scores to dictate escalation of therapy would lead to better outcomes than using easily administered clinical scores such as the DAS28_CRP_. While clinical trials of bDMARDs including golimumab [14] and tocilizumab [15] indicated that clinical responses were associated with improvements in MRI inflammation scores, there is little information comparing clinical and MRI responses to cDMARDs in the T2T “real-world” setting.

The aim of this study was to compare the response in clinical disease activity (DAS28_CRP_) with response in MRI inflammation scores after T2T-dictated escalation of therapy in two groups of RA patients: those escalating from cDMARDs to a different cDMARD combination and those escalating to a combination that includes anti-TNF therapy.

## Methods

### Power calculation

Studies exploring the relation between ΔMRI synovitis scores and ΔDAS28_CRP_ [7, 16, 17] indicated a simple correlation of 0.4. Using a two-sided test at the 5% significance level with 80% power, the required total sample size would be 46 patients. Allowing for 10% dropout, the estimated sample size required was 50. For comparing MRI(i) responses between patients escalating to cDMARDs versus cDMARD/anti-TNFs, sample size was calculated based on the one-sided hypothesis that triple therapy would not be inferior to MTX/anti-TNF. We estimated that with 50 patients per group the study would have 80% power at 5% significance to establish non-inferiority, assuming a between-group difference in change from baseline to 3 months of 2 (SD = 2). Although enrolment was continued for 3 years, only 80 patients with seropositive RA were recruited from rheumatology outpatients’ clinics from Auckland and Christchurch, New Zealand between 2014 and 2016. Two cohorts were recruited: Group A included patients on cDMARD therapy (either methotrexate alone or a cDMARD combination), escalating to a different cDMARD combination; and Group B included patients on a cDMARD combination who were escalating to bDMARDs including an anti-TNF agent. Patients were required to have a DAS28_CRP_ ≥ 3.2 at the first clinical visit. Tender joint counts (28 and 68 joints) and swollen joint counts (28 and 66 joints) were performed by trained research nurses and patients filled out questionnaires to assess function. Serum was obtained for inflammatory markers and rheumatoid serology. Written informed consent was obtained from all patients. The study was approved by the Health and Disability Ethics Committees, New Zealand.

### MRI scans and scoring

The dominant wrist was scanned using a 3 T scanner (Philips MR Systems Achieve 3 T; Koninklijke Philips Electronics NV, Eindhoven, the Netherlands). An eight-element Philips SENSE 3.0 T Wrist Coil 8 Channel (Invivo Corp, Gainsville, FL, USA) (receive only) was used. The dominant hand in the wrist coil fitted snugly by the patient’ s side, palm to body, thumb anteriorly. The field of view included the carpus, distal radioulnar joint (dRUJ) and metacarpal (MC) bases. The following were used: turbo spin echo sequences, T1 weighted (T1w) and T2 weighted (T2w) with fat saturation (FS) using spectral adiabatic inversion recovery (SPAIR) in axial and coronal planes and proton density (PD) coronals (without FS). T1wFS axial and coronal sequences were obtained post intravenous gadolinium diethylenetriamine pentaacetic acid (GdDTPA) at standard dosage (Omniscan (Gadodiamide; 5.0 mmol/10 ml or 2.87 g/10 ml); GE Healthcare, Inc., Princeton, NJ, USA). Synovitis, osteitis and erosions were scored according to RAMRIS [8] and tenosynovitis in the flexor and extensor compartments [9]. MRI scan pairs (baseline, 4 months) were scored in known chronological order by one experienced reader (AD), blinded to clinical and X-ray data. Ten scans were selected randomly and rescored 6 months later. The intra-reader reliability was high: ICC (av) = 0.89 (99% CI 0.56–0.97).

### X-rays

Plain radiographs of the hands and feet were obtained for all patients on enrolment. They were scored for damage using the Sharp van der Heijde (SvdH) score [18] by a rheumatologist (ND), blinded to clinical and MRI data.

### Statistical analysis

Intra-reader reliability for MRI scores was determined using intraclass correlation coefficients (ICCs). Spearman’s correlations were used to examine the association between ΔDAS28_CRP_ and ΔMRI scores. Clinical characteristics for Groups A and B were compared using the chi-square test and median two-sample test. The effect of ΔDAS28_CRP_ and treatment group on ΔMRI(i) were analysed using multiple linear regression adjusting for age, gender, duration, anti-CCP titre and MRI erosion score at baseline. Four patients transited from Group A into Group B, providing eight scan pairs. Apart from analyses investigating baseline patient characteristics where the four patients were included only in their initial Group A, all analyses were performed including these patients in both groups and were conducted using SAS for Windows version 9.4 (SAS Institute Inc., Cary, NC, USA).

## Results

**Fig. 1:**
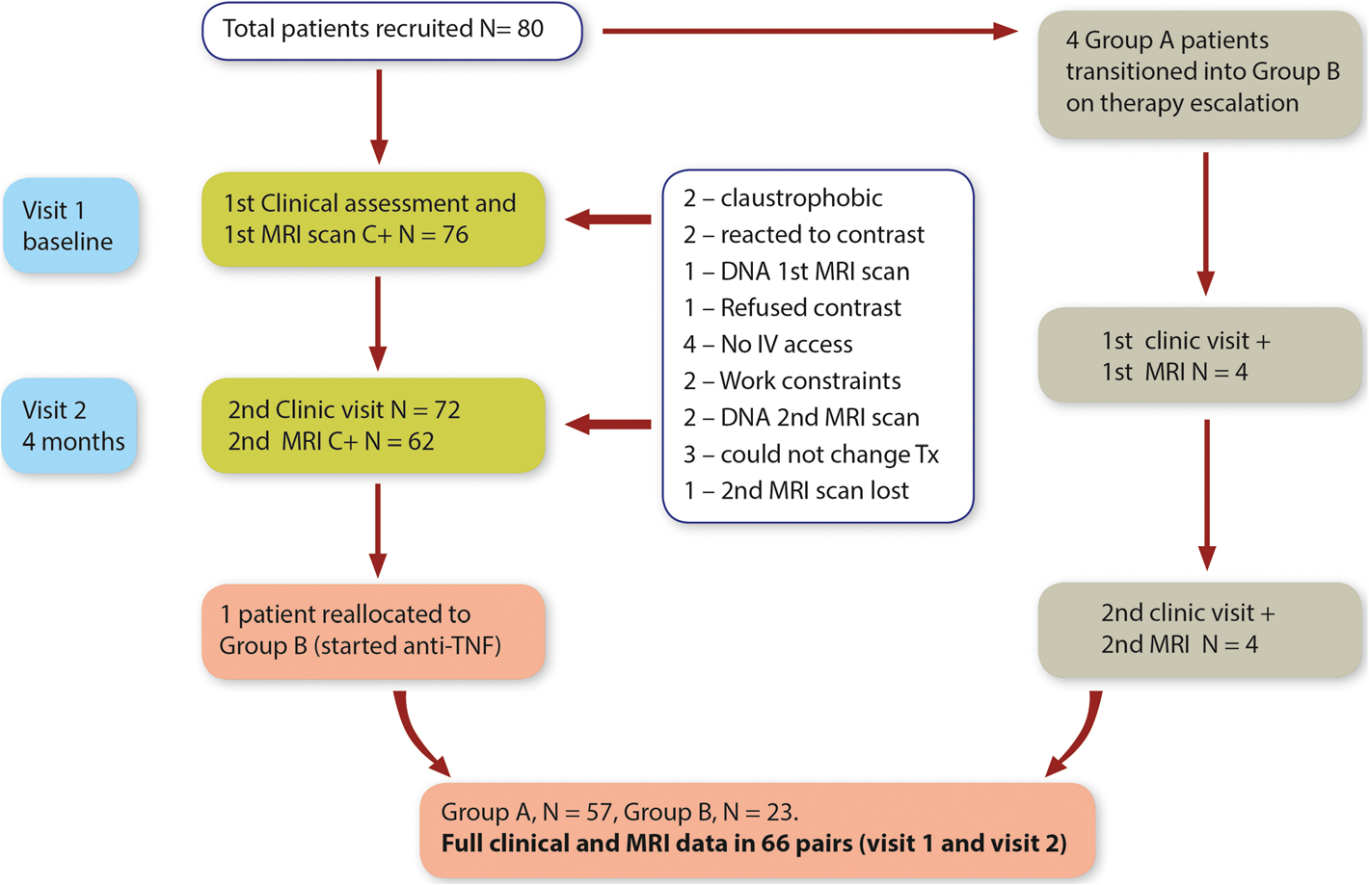
Clinical and MRI data gathered as patients progressed through the study. IV intravenous, MRI magnetic resonance imaging, TNF tumour necrosis factor, Tx therapy

A total of eighty patients were enrolled, fifty-seven in Group A (42 female, 15 male) and 23 patients in Group B (5 female, 18 male) [5, 18]. Dropout throughout the protocol is summarized in Fig. 1. One patient began adalimumab for disease control 1 month after enrolment and remained on this bDMARD for 3 months before the second MRI scan and so was switched to Group B. Full clinical and contrast-enhanced MRI data from Visit 1 (baseline) and Visit 2 (4 months) were available for 62 patients. However, 66 scan pairs were available for analysis (including eight pairs from four patients who were sequentially enrolled into Group A then Group B).

### Clinical characteristics

Patients in Group A and Group B were of similar age and gender but those in Group B had a longer disease duration (Table 1). Consistent with their different places on the T2T escalation ladder, swollen and tender joint counts and disease duration (Visit 1) were lower in Group A than Group B (Table 2). The DAS28_CRP_ was also lower for Group A than Group B: 4.24 vs 5.15 (*p* = 0.007). Interestingly, there was no difference between groups for MRI synovitis or tenosynovitis scores at Visit 1 but total MRI inflammation and osteitis scores were lower in Group A than Group B (*p* = 0.05 and 0.02 respectively) (Table 2). MRI erosion scores were also lower in Group A than Group B: 4 (0–50) and 9 (1–47) respectively (*p* = 0.03). The total SvdH X-ray damage score (Visit 1) was also lower in Group A than Group B: 20.5 (0–217) and 63 (0–209) respectively (*p* = 0.03).

**Table 1:** Patient characteristics on enrolment

Clinical characteristic at recruitment	Total		Group A		Group B		Chi-square *p* value*
	(*N* = 80)		(*N* = 57)		(*N* = 23)		
Gender							
Female	60	75%	42	73.68%	18	78.26%	0.67
Male	20	25%	15	26.32%	5	21.74%	
Age (months)	54.5 (24, 82)		53 (24, 81)		56 (27, 82)		0.22
Disease duration (months)	36 (3, 408)		30 (3, 408)		72 (15, 240)		0.0014
On methotrexate							
Yes	70	87.5%	49	85.96%	21	91.3%	0.72
No	10	12.5%	8	14.04%	2	8.7%	

Data presented as *n* (%). Age and disease duration expressed as median (minimum, maximum) values

*Median two-sample test *p* value

**Table 2:** Clinical and imaging scores at Visit 1 and Visit 2 (4 months later)

	Total (*N* = 84)				Group A (*N* = 57)				Group B (*N* = 27*)				Median two-sample test *p* value
	*N*	Median	Minimum	Maximum	*N*	Median	Minimum	Maximum	*N*	Median	Minimum	Maximum	
Clinical scores at Visit 1													
Tender 28 joints	84	11	1	28	57	10	1	28	27	14	1	28	0.050
Swollen 28 joints	84	4	0	20	57	4	0	11	27	6	0	20	0.097
HAQ	84	1.06	0	2.5	57	0.88	0	2.13	27	1.38	0.13	2.5	0.037
DAS28_CRP_	84	4.5	3.24	6.64	57	4.24	3.24	6.04	27	5.15	3.36	6.64	0.003
MRI scores at Visit 1													
Synovitis	77	3	0	9	51	3	0	9	26	4	0	9	0.102
Tendonitis	77	3	0	15	51	3	0	15	26	3	0	15	0.931
Osteitis	77	2	0	34	51	2	0	34	26	3	0	24	0.021
Erosion	76	5	0	50	50	4	0	50	26	9	1	47	0.030
Total inflammation	77	9	0	45	51	8	0	45	26	13	1	36	0.050
X-ray scores at Visit 1													
Erosions	62	13	0	130	42	5.5	0	112	20	33.5	0	130	0.001
Total SvdH score	62	35.5	0	217	42	20.5	0	217	20	63	0	209	0.031
Clinical scores at Visit 2													
Tender 28 joints	72	6	0	28	48	5	0	28	24	7	0	24	0.399
Swollen 28 joints	72	1	0	20	48	1	0	20	24	0.5	0	9	0.337
HAQ	72	0.63	0	2.13	48	0.5	0	2	24	0.75	0	2.13	0.155
DAS28_CRP_	72	3.47	1.42	6.64	48	3.28	1.42	6.64	24	3.66	1.42	5.49	0.047
MRI scores at Visit 2													
Synovitis	66	3	0	9	42	3	0	9	24	3	0	8	0.851
Tendonitis	66	2.5	0	15	42	2	0	15	24	3	0	12	0.612
Osteitis	68	2	0	34	44	2	0	32	24	3.5	0	34	0.102
Erosion	67	6	0	50	43	4	0	50	24	9.5	1	47	0.009
Total inflammation	66	9	1	53	42	9	1	43	24	9	1	53	1.000

*Includes the four patients who transitioned from Group A to Group B on escalation of therapy (see text)

*DAS28*_*CRP*_ disease activity score (28 joints) incorporating C-reactive protein, *MRI* magnetic resonance imaging, *SvdH* Sharp van der Heijde, *HAQ* Health assessment questionnaire

### Changes in clinical disease activity and MRI inflammation on escalation

Patients’ responses were measured by ΔDAS28_CRP_. Both groups improved from Visit 1 to Visit 2 so that ΔDAS28_CRP_ for Group A was −0.94 (−3.30, 1.61) and for Group B was −1.53 (−3.59, 0.56). There was no difference between groups (*p* = 0.45). For Groups A and B, 83.3% and 87.5% respectively had a fall in the DAS28_CRP_ from Visit 1 to Visit 2 indicating clinical improvement, while in 16.7% and 12.5% there was worsening. ΔMRI(i) from Visit 1 to Visit 2 varied between patients but median scores showed little change over 4 months, despite escalation of therapy, and there was no difference between groups. For Group A, ΔMRI(i) median (range) was 0 (−25, 10); for Group B, −1 (−15, 28) (*p* = 0.12). Table 3 shows how patients responded in terms of MRI inflammation (total and sub-scores) and MRI erosion score. Only a few showed MRI erosion progression (three patients (6.98%) in Group A and five patients (20.83%) in Group B). The majority had no change in MRI erosion scores (93.0% and 79.17% respectively).

**Table 3:** Direction of change for MRI scores from Visit 1 to Visit 2

					Total
	Group A		Group B		
	*n*	%	*n*	%	
Change in MRI(i)					
Decrease	16	38.1	13	54.17	29
Increase	16	38.1	3	12.5	19
No change	10	23.81	8	33.33	18
Total	42		24		66
Change in MRI osteitis					
Decrease	15	34.09	8	33.33	23
Increase	9	20.45	6	25	15
No change	20	45.45	10	41.67	30
Total	44		24		68
Change in MRI synovitis					
Decrease	15	35.71	11	45.83	26
Increase	9	21.43	1	4.17	10
No change	18	42.86	12	50	30
Total	42		24		66
Change in MRI tenosynovitis					
Decrease	12	28.57	9	37.5	21
Increase	10	23.81	5	20.83	15
No change	20	47.62	10	41.67	30
Total	42		24		66
Change in MRI erosion					
Increase	3	6.98	5	20.83	8
No change	40	93.02	19	79.17	59
Total	43		24		67

*MRI* magnetic resonance imaging, *MRI(i)* total MRI inflammation score at the wrist (sum of synovitis, osteitis and tendonitis scores)

**Fig. 2:**
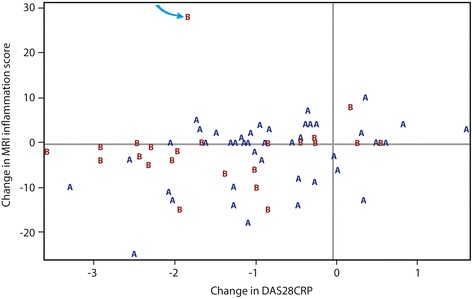
Change in MRI inflammation score (over 4 months) plotted against change in DAS28_CRP_ for each individual patient (labelled A or B according to group). Arrow points to an outlier in Group B (see text). DAS28_CRP_ disease activity score (28 joints) incorporating C-reactive protein, MRI magnetic resonance imaging

Combining groups, there was a weak positive correlation between ΔMRI(i) and ΔDAS28_CRP_ (Spearman’s 0.36, *p* = 0.003). Only ΔMRI tenosynovitis correlated with ΔDAS28_CRP_ (Spearman’s 0.33, *p* = 0.007); MRI sub-scores for Δosteitis, Δsynovitis and Δerosions did not (Table 4). Multiple linear regression analysis was used to assess the effect of group and ΔDAS28_CRP_ on ΔMRI(i) adjusting for age, gender, duration, anti-CCP titre and MRI erosion score at Visit 1. ΔDAS28_CRP_ was associated with ΔMRI(i) so that for every unit increase in ΔDAS28_CRP_ there was a 1.77-U increase in ΔMRI(i) (*p* = 0.056). Findings are summarized in Fig. 2.

**Table 4:** Correlations between ΔDAS28_CRP_ and ΔMRI scores

Change in MRI scores (over 4 months)	Number of observations	Spearman correlation coefficients	*p* value
Δtotal MRI(i)	66*	0.36	0.003
Δosteitis	68	0.12	0.33
Δsynovitis	66	0.21	0.09
Δtendonitis	66	0.33	0.007
Δerosion	67	–0.02	0.87

*Sixty-six scan pairs available, includes eight pairs from the four patients who transitioned from Group A to Group B

*DAS28*_*CRP*_ disease activity score (28 joints) incorporating C-reactive protein, *MRI* magnetic resonance imaging, *MRI(i)* total MRI inflammation score at the wrist (sum of synovitis, osteitis and tendonitis scores)

### Change in MRI score did not differ between groups

There was no difference between groups for ΔMRI(i) although the point estimate of the fall in score was numerically greater for Group A (−3.68 (95% CI −6.16 to − 1.20)) than Group B (−0.84 (95% CI −4.27 to 2.60), *p* = 0.18). We did note an outlier in Group B in whom there was a significant fall in the DAS28_CRP_ (from 5.15 (V1) to 3.31 (V2)) despite a marked rise in MRI inflammation scores (from 25 to 53). Data for this patient were excluded in a sensitivity analysis but the difference between groups remained non-significant (*p* = 0.27).

### MRI erosion score at V1 was associated with change in MRI(i)

The MRI erosion score (V1) was negatively associated with ΔMRI(i) (parameter estimate −0.28; *p* = 0.0052), so those with higher MRI erosion scores at V1 had a greater fall in MRI(i) from V1 to V2. For every 1-U increase in the V1 erosion score, the MRI(i) score would be estimated to fall from V1 to V2 by 0.28 U. The X-ray erosion score (V1) was not associated with ΔMRI(i) (*p* = 0.79).

## Discussion

This is the first real-world study to investigate the link between changes in clinical disease activity and imaging (MRI) joint inflammation, in a cohort of RA patients undergoing escalation of therapy according to T2T principles. In this setting, the clinical target is most often a low disease activity state or complete remission (DAS28_CRP_ ≤ 1.9). It has been suggested that more stringent imaging targets should be used so that patients are treated aggressively, aiming to reach a state where there is no imaging evidence of inflammation at all [19]. MRI scanning can inform about inflammation affecting different tissues including synovium (synovitis), bone (osteitis) and tendon sheaths/tendons (tenosynovitis). We found a significant but relatively weak association between change in clinical disease activity as measured by the DAS28_CRP_ over the 4-month period after escalation of therapy, and change in the total MRI inflammation score. The only MRI sub-score separately associated with ΔDAS28_CRP_ was the ΔMRI tendonitis score, although there was a trend towards an association with the ΔMRI synovitis score. These findings are consistent with observations by Krabben et al. [11], who found in their analysis of 1790 joints in 179 patients that synovitis and tenosynovitis on MRI were independently associated with clinical swelling. In that study, MRI inflammation was most often observed at the wrist, validating our choice of site for MRI scanning.

Although we did observe an association between change in clinical disease activity and change in MRI inflammation scores, it was obvious from inspection of the data that many individual patients improved clinically over the 4 months after treatment escalation, with only minor changes in their MRI inflammation scores. In some instances MRI inflammation worsened despite clinical improvement. Could this be due to the much greater sensitivity of MRI for detecting joint inflammation? Clinicians are familiar with high-grade clinical joint inflammation manifesting as Virchow’s triad of redness, swelling and tenderness, while lesser degrees may only be detectable as pain on movement. MRI can detect inflammation below the clinical threshold by revealing thickened synovium and tenosynovium, which often enhance post contrast, reflecting increased vascularity. However, if MRI inflammation simply represents the low end of the inflammatory spectrum, one might expect a closer correlation between clinical and imaging observations than we have found. A ceiling effect for MRI inflammation scores could be part of the explanation. Thus, a severely inflamed wrist joint might achieve a very high MRI inflammation score, which remains high even when joint swelling and tenderness have subsided. A period of 4 months might also have been insufficient time for MRI(i) to fall in some patients [20]. Another explanation for the relative disparity between changes in clinical and MRI scores is that they capture slightly different information. Clinical disease activity scores incorporate (through joint tenderness) an assessment of pain and this is missing from the MRI inflammation score. The MRI inflammation score incorporates osteitis, which is not directly detectable clinically. Additionally, low-grade synovitis may occur in normal individuals, especially older people [21], and this could contribute to background “noise” within the MRI inflammation score. In this study we have used the DAS28_CRP_ as a “gold standard” against which changes in MRI inflammation were compared, but this measure has its own shortcomings and in some studies the physician global assessment is a better predictor of clinical decision-making [22].

Another possible explanation for the relatively poor correlation between change in MRI inflammation scores and change in the DAS28_CRP_ might be that MRI scans in this study imaged one region only, the dominant wrist, while the DAS28_CRP_ reflected disease activity at 28 joints. We were able to retrieve joint count data and found evidence for tenderness and/or swelling at the scanned wrist in 71.2% of patients at Visit 1 and 47% at Visit 2. Thus, the wrist was quite frequently involved. How well do MRI scans of the wrist reflect, or in other words act as a surrogate marker for, overall rheumatoid disease activity? The evidence for this is indirect. In an early study by McQueen et al. [12], MRI wrist bone oedema (osteitis) scores were predictive of radiographic erosion progression at the hands and feet 6 years later. In 2014, Baker et al. [20] reported a very similar result for MRI osteitis and synovitis from one hand (wrist and MCPs), which were again highly predictive of radiographic progression in the joints of the hands and feet 1 and 2 years later. In 2017, Glinatsi et al. [23] published a post-hoc analysis of clinical trial data from the CIMESTRA/OPERA cohorts and concluded that MRI-assessed inflammation at the wrist (rather than at the MCP joints) was the most important parameter reflecting physical function, pain and global assessment of disease activity in early RA. Thus, although MRI scanning of one joint area may potentially suffer from “sampling error”, there is evidence that the wrist is a particularly informative region.

One of the aims of the study was to compare MRI parameters between patients escalating from one cDMARD combination to another cDMARD combination (Group A) and patients escalating to anti-TNF therapy/cDMARDs (Group B). Group B patients, who were further up the T2T escalation ladder, differed from Group A patients in that they had higher scores for MRI erosion and osteitis at their first visit. Interestingly, there were no differences between groups in terms of synovitis or tendonitis. This is consistent with the known association between MRI osteitis and erosion progression in RA [12, 13], and emphasizes that bone inflammation (osteitis) may be qualitatively and prognostically different from soft tissue joint inflammation (synovitis and tenosynovitis). Molenaar et al. [24] observed that clinically relevant damage progression can occur in patients in prolonged remission. Thus, MRI may have an important role in the detection of osteitis and prediction of damage progression; a role that cannot be duplicated by clinical assessment alone. Very few of our patients demonstrated MRI erosion progression over 4 months but longer follow-up would be required to determine whether those with clinically normal joints but high levels of osteitis had a worse erosive outcome. We did not find evidence for a greater reduction in MRI inflammation scores in those receiving anti-TNF therapy than those on cDMARDs. Although Groups A and B cannot be directly compared because of differences in disease duration, this supports the observations of O’Dell et al. [25] who showed equivalent clinical improvement in patients treated with triple therapy and those receiving anti-TNF/methotrexate therapy. Our findings also go some way towards validating the NZ PHARMAC escalation protocol that requires patients to have first tried cDMARD therapy before escalating to more costly bDMARD options.

What are the implications of our findings for the treating clinician? T2T regimens using clinical targets have been profoundly effective in limiting the progression of joint damage and disability in RA patients on cDMARD and bDMARD regimens [26]. If the goal posts are shifted to more stringent imaging targets, what is the evidence for better outcomes? The OMERACT group have studied this by looking for evidence of a “safe level” of MRI inflammation below which damage progression did not to occur, using MRI data from several RA clinical trial cohorts [27]. Gandjbakhch et al. identified an “acceptable MRI inflammatory activity state” as a RAMRIS synovitis score below 6. In these individuals, radiographic progression rarely occurred. As yet there is no evidence that clinical or functional outcomes are better in such patients. Our study has indicated considerable disparity between clinical disease activity and MRI joint inflammation, and has emphasized that the goal of complete imaging resolution of inflammation is far off in the majority of real-world patients. Is it appropriate to treat these individuals more aggressively? Issues of feasibility, cost and patient acceptability mean that clinical goals are probably to be preferred over MRI goals at least in the short term. Whether ultrasound can step into the gap remains an open question [28]. Both the ARCTIC [29] and the TaSER [30] studies have recently investigated whether ultrasound imaging can improve patient outcomes if used by clinicians to inform decision-making. Both studies concluded that an ultrasound-driven T2T strategy was not associated with significantly better clinical or imaging outcomes than a DAS28-driven strategy. Further large prospective studies of this type are now needed for MRI scanning.

Our study has a number of limitations. Firstly, the study was observational. It was not possible to stipulate what disease-suppressing therapies could be given and the associations we observed could have been influenced by individual DMARDs. We considered stratifying patients according to therapy but there were many combinations of cDMARDs used and groups would have been too small for useful comparison. However, we did establish that a similar proportion of Group A and Group B patients were taking methotrexate at their first visit. Secondly, although we found no difference between groups in clinical or MRI responses to therapy, this comparison was flawed by insufficient statistical power and also by the fact that the groups differed in terms of disease duration and radiographic erosions at baseline [31]. Despite extending enrolment by a year we did not reach the numbers required to satisfy the original power calculation. Nevertheless, our comparative clinical and MRI data do shed light on what is actually happening in terms of joint inflammation in rheumatoid patients undergoing escalation of therapy in routine rheumatology practice, and should be of help to clinicians working towards the aim of improving quality of life in their patients.

## Conclusions

Full clinical and MRI data were available at two time points, before and 4 months after a change of therapy, in 66 out of 80 RA patients undergoing real-world T2T escalation. There was no or minimal change in the median MRI inflammation score over 4 months, despite clinical improvement. There was no difference in the MRI inflammation response between those escalating to a different cDMARD regimen and those escalating to a cDMARD/anti-TNF regimen. For the entire group there was a significant correlation between the change in the DAS28_CRP_ and the change in the MRI inflammation score (Spearman’s 0.36, *p* = 0.003), but this was relatively weak. These results suggest that MRI outcomes should not be adopted to direct T2T escalation as clinically driven strategies are already successful in improving patient outcomes. A randomized controlled trial comparing MRI-driven and DAS-driven strategies is needed.

## Abbreviations

3T-MRI: 3-Tesla magnetic resonance imaging
Anti-CCP: Anti-cyclic citrullinated peptide antibodies
bDMARD: Biological disease-modifying therapy
cDMARD: Conventional disease-modifying therapy
CRP: C-reactive protein
DAS28_CRP_: Disease activity score (28 joints) incorporating CRP
dRUJ: Distal radioulnar joint
FS: Fat saturation
GdDTPA: Gadolinium diethylenetriamine pentaacetic acid
ICC (av): Intraclass correlation coefficient (average)
MC: Metacarpal
MRI(i): Total MRI inflammation score at the wrist (sum of synovitis, osteitis and tendonitis scores)
MTX/anti-TNF: Methotrexate combined with anti-tumour necrosis therapy
OMERACT: Outcome Measures in Rheumatology
PD: Proton density
PHARMAC: Pharmaceutical Management Agency (New Zealand)
RA: Rheumatoid arthritis
RAMRIS: Rheumatoid Arthritis Magnetic Resonance Imaging Score
SAS: Statistical Analysis System
SPAIR: Spectral adiabatic inversion recovery
SvdH: Sharp van der Heijde
T1w: T1 weighted
T2T: Treat to target
T2w: T2 weighted
V1: Visit one
V2: Visit two
ΔDAS28_CRP_: Change in the DAS28_CRP_
ΔMRI(i): Change in the total MRI inflammation score (wrist)
